# Acetylation Modification of Waste Polystyrene and Its Use as a Crude Oil Flow Improver

**DOI:** 10.3390/polym13152505

**Published:** 2021-07-29

**Authors:** Wangyuan Zhang, Michal Slaný, Jie Zhang, Yifan Liu, Yunlei Zang, Yongfei Li, Gang Chen

**Affiliations:** 1 State Key Laboratory of Petroleum Pollution Control, Xi’an Shiyou University, Xi’an 710065, China; 19211070698@stumail.xsyu.edu.cn (W.Z.); zhangjie@xsyu.edu.cn (J.Z.); 20211070810@stumail.xsyu.edu.cn (Y.L.); zyl1010204796@hotmail.com (Y.Z.); b16020082@s.upc.edu.cn (Y.L.); 2 Institute of Inorganic Chemistry, Slovak Academy of Sciences, Dúbravská cesta 9, 845 36 Bratislava, Slovakia; 3 Institute of Construction and Architecture, Slovak Academy of Sciences, Dúbravská cesta 9, 845 03 Bratislava, Slovakia; 4 Shaanxi Province Key Laboratory of Environmental Pollution Control and Reservoir Protection Technology of Oilfields, College of Chemistry and Chemical Engineering, Xi’an Shiyou University, Xi’an 710065, China

**Keywords:** heavy oil, flow improver, waste polystyrene, pour point, viscosity

## Abstract

Polystyrene is used in a wide range of applications in our lives, from machine housings to plastic cups and miniature electronic devices. When polystyrene is used, a large amount of waste is produced, which can cause pollution to the environment and even harm biological and human health. Due to its low bulk density (especially the foamed type) and low residual value, polystyrene cannot be easily recycled. Often waste polystyrene is difficult to recycle. In this paper, waste polystyrene has been modified by using acetic anhydride which caused a crude oil flow improver. The results showed that modified polystyrene improves the flow properties of the crude oil, reducing the viscosity and the pour point of the crude oil by up to 84.6% and 8.8 °C, respectively. Based on the study of the paraffin crystal morphology, the mechanism of improving the flow capacity of crude oil by modified polystyrene was proposed and analyzed.

## 1. Introduction

Polystyrene can be applied as a material for many purposes (e.g., masks, filter membranes, etc.) which are using in our daily life. The annual consumption of polystyrene for polystyrene foam manufacturing in China is around 1.5 million tons, and its consumption increasing at an annual rate of 10%. Waste from polystyrene is highly stable and does not readily degrade naturally. For this reason, the use of conventional methods, e.g., landfilling and incineration, to dispose of polystyrene foam waste will cause not only environmental pollution but waste resources, too. Therefore, waste polystyrene must be properly disposed of. On the other hand, the recycling of waste polystyrene can solve not only the problem of environmental pollution causing by its combustion, but also achieve the effect of energy saving and emission reduction.

Crude oil is a very complex system that consists of many hydrocarbon and non-hydrocarbon compounds. In general, Chinese crude oil is characterized by a low recovery of light oil and a high wax content. During the transportation of crude oil, the increasing of crude-oil wax content can cause blockages in the pipelines [1,2]. In the case of temperature decreases, the waxes in the crude oil will crystallize and form aggregates, i.e., 3D spatial network structures that increase the adhesion of the crude oil which affects its production and transportation [3,4]. In order to improve the fluidity of crude oil with high wax content, many solutions have been developed. However, in general, these methods have problems with high energy consumption as well as a high investment cost. The addition of flow improvers to crude oil is considered as the most convenient and most economical way to improve its flowability [5,6].

**Figure 1:**
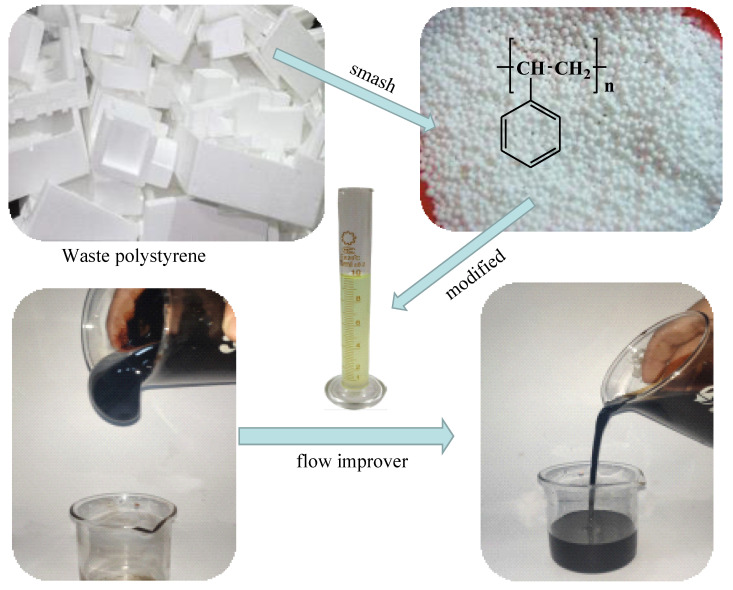
Application of waste polystyrene in viscosity reduction of crude oil.

At present, the flow improvers can be split into several types, surfactants, and polymers, etc. [7]. The addition of surfactants leads to the formation of oil-in-water emulsion and hence significantly reduces the viscosity of production liquids in the pipelines [8,9]. However, there are still problems with the selective nature of thick oil, the difficulty of treating effluent from the recovery fluid, and the fact that various methods of breaking emulsions and dewatering still exist. Polymers are typically chemical additives that act according to their structure and composition. Commonly used crude oil flow improvers include EVA pour point reducer, ECA-814 flow improver, PLC-102 and A-137 pour point reducer [10]. The synthesis of acetylated modification of polystyrene is highly chemically and thermally stable due to its chemical nature. On the other hand, the acetylation modification process for waste polystyrene is simple and easy to apply in a wide range of applications. In this paper, a crude oil flow improver was prepared by the modification of waste polystyrene using acetic anhydride and the performance of the modified polystyrene was evaluated. The modified polystyrene may disrupt the formation of wax crystals and promote the flowability of the crude oil. It can be expected that the proposed ideas (Figure 1) contribute to recycle and reuse the wasted polystyrene products, which would also benefit environmental protection.

## 2. Materials and Methods

### 2.1. Material

Acetic anhydride was obtained from Nanjing Chemical Reagent Co., Ltd. (Nanjing, China), and pyridine was purchased from Sinopharm Chemical Reagents Co., Ltd. (Shanghai, China). The 0# diesel oil was originated from Sinopec. Waste polystyrene was obtained from foam boards which were used in boxes by cutting into fine particles up to 3 mm in diameter. The crude oil samples are from Henan Oilfield of China. The basic physical parameters of obtained samples were listed in Table 1.

### 2.2. Modification of Polystyrene

**Figure 2:**
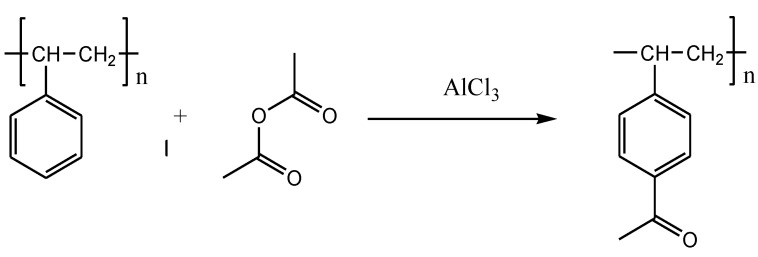
Modification of polystyrene by acetylation.

The waste polystyrene was put into a single-neck flask to dissolve with a certain amount of pyridine solution. Subsequently, polystyrene and acetic anhydride were mixed with a molar ratio of 1:1. The single-neck flask was placed in a thermostatic heating sleeve. The reaction (Figure 2) was kept at 115 °C for 4 h. After the reaction, the mixed solution was filtered and purified to obtain the required product. The acetylated polystyrene was dissolved in diesel with different proportions, to obtain the oil-soluble viscosity depressant with an optimized polymer-diesel ratio.

### 2.3. FT-IR Characterization

The modified polystyrene was characterized by (FT-IR) Fourier Transform Infrared Spectroscopy (Thermo Electron Co., Waltham, MA, USA). The method was utilized with the liquid membrane method, in which the sample was directly coated into thin liquid film and placed in an optical path for detection. Wavenumber range from 4000 cm^−1^ to 400 cm^−1^ with a spectral resolution of 4 cm^−1^ and 64 scans was used for measurement [11].

### 2.4. Viscosity Measurement

The viscosity of the treated heavy oil was determined using a BROOKFIELD DV-II + programmable Viscometer at certain temperatures according to Industrial Standard of China Petroleum, SY/T0520-2008. For each measurement, 30 g of crude oil was poured into a measuring cup and heated up to 50 °C. Crude oil flow improver obtained by dissolving modified polystyrene in different volumes of diesel oil was added to the crude oil at different concentrations (100–1000 mg/L). After that, the mixture was properly vortexed and the crude oil viscosity was measured at different temperatures.

### 2.5. Pour Point Test

During each experiment, different concentrations (100–1000 mg/L) of crude oil flow improver were added to the crude oil. After that, obtained mixtures were properly vortexed, and then the pour points were measured according to SY/T0541-2009.

### 2.6. Optical Microscopy Analysis

The saturated hydrocarbon was separated from crude oil by using the method in accordance with SY/T 5119-2016. The influence of crude oil flow improver on the morphology of wax crystal was observed by polarizing microscope (BX41-OLYMPUS, Japan). During each measurement, the sample was firstly kept at 50 °C to eliminate the wax crystals. Subsequently, the sample was cooled down to 15 °C within 5 min. The formation and morphology of the wax crystals were investigated. An external thermostat was used to keep the temperature at 15 °C.

## 3. Results

### 3.1. IR Spectrum Analysis

**Figure 3:**
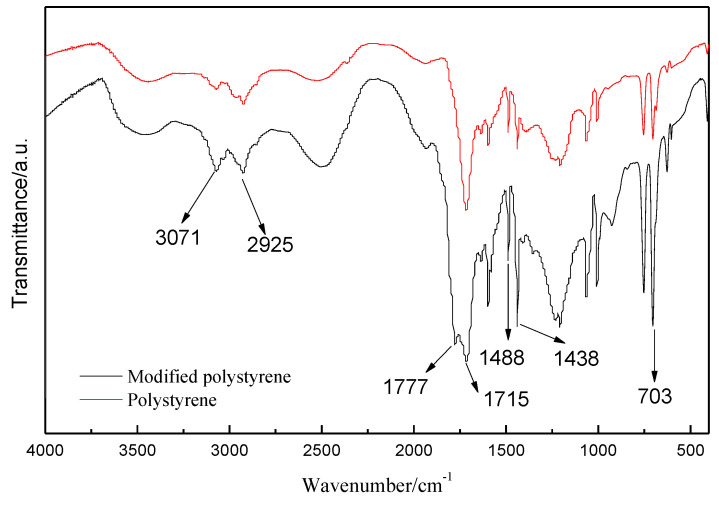
FT−IR spectrum analysis of modified polystyrene.

The IR characterization was used to confirm the functional groups of the modified polystyrene. The obtained spectra were shown in Figure 3. The IR spectrum of the modified polystyrene shows absorption bands at 1438 cm^−1^ and 1488 cm^−1^, which are assigned to the C=C vibration. The absorption peaks at 1715 cm^−1^ and 1777 cm^−1^ are assigned the symmetric and antisymmetric stretching vibration of C=O groups in acetic anhydride. In addition, the absorption peaks at 703 cm^−1^ are the monosubstituted group of the benzene ring. Finally, the absorption bands at 2925 cm^−1^ and 3071 cm^−1^ are the characteristics for the CH_3_ and CH_2_ groups, respectively. The characteristic peaks are basically consistent with the standard spectra of modified polystyrene by acetic anhydride. The appearance of a new peak at 1777 cm^−1^ in the IR spectrum of the modified polystyrene in comparison with the IR spectrum of polystyrene indicates a successful acetylation modification of the polystyrene [12].

### 3.2. Effect of Mixed Solvents on Viscosity and Pour Point

**Figure 4:**
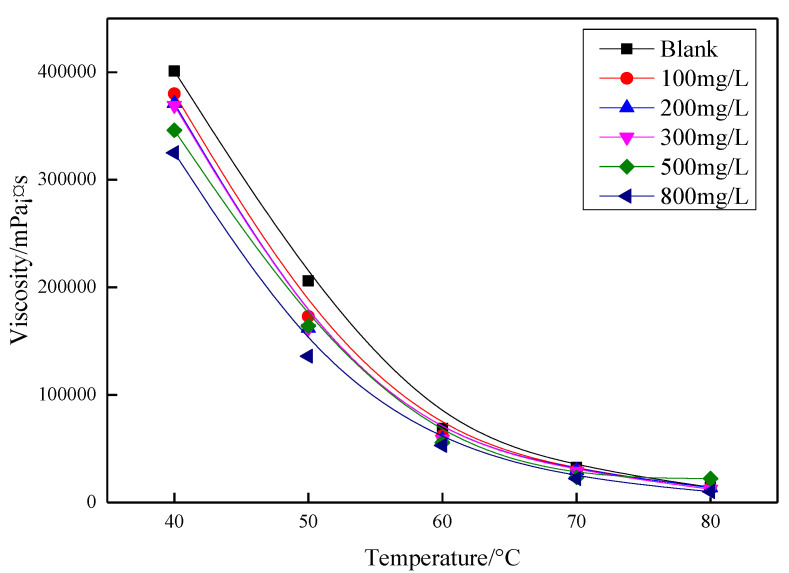
Effect of diesel-pyridine solvent blends on the viscosity of oil samples.

Pyridine and diesel are mixed in a 1:1 ratio by volume to obtain a pyridine-diesel mixed solvent. The effect of the solvent mixture without modified polystyrene on the viscosity and pour point of the oil samples was determined by adding different concentrations of the pyridine-diesel mixture to the measuring cup containing the crude oil. The effects of different concentrations of pyridine-diesel mixed solvents on the viscosity and pour point of the oil sample were shown in Figure 4 and Table 2. From Figure 4, it can be seen that pyridine-diesel blends without modified polystyrene do not have a significant effect on the viscosity and pour point of Henan oil samples. The modified polystyrene was dissolved in a pyridine-diesel solvent mixture for better fluidity performance. Based on the results, the influence of solvent can be eliminated, and the effect of modified polystyrene can be better recognized.

### 3.3. Dissolution Effect of Reaction Product and Diesel Oil

**Figure 5:**
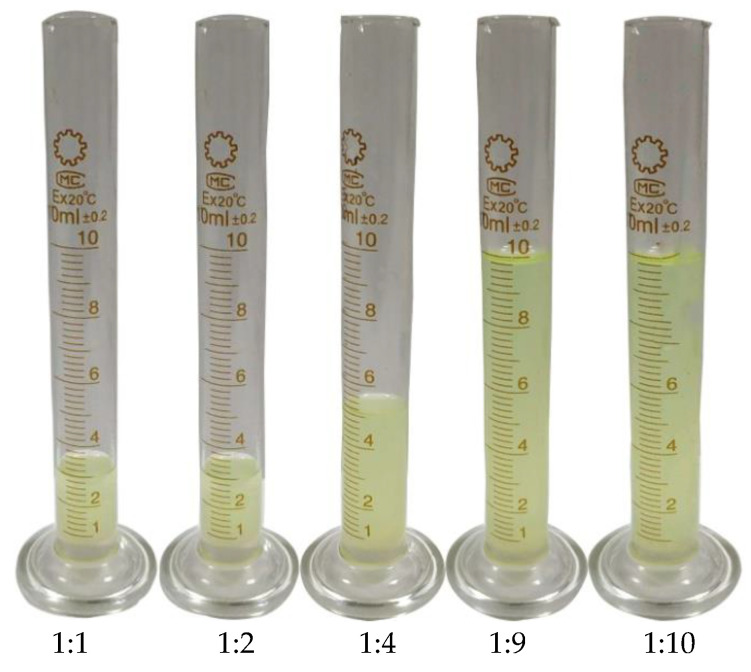
Mixing effect of different volume ratios of reaction products and diesel.

The modified polystyrene was dissolved in diesel with different concentrations and the dissolution effect was observed. From Figure 5, it can be seen that the reaction products were adequately soluble within different volume ratios of diesel. In addition, all the mixed solutions were filtered and centrifuged, and no precipitation was found. Therefore, five different concentrations of oil-soluble solutions (named COFI-1 COFI-2 COFI-3 COFI-4 COFI-5) were used as oil-soluble viscosity. Coagulation reducers to evaluate the effect of viscosity and coagulation reduction of the oil samples were performed [13].

### 3.4. Viscosity Reduction Performance

**Figure 6:**
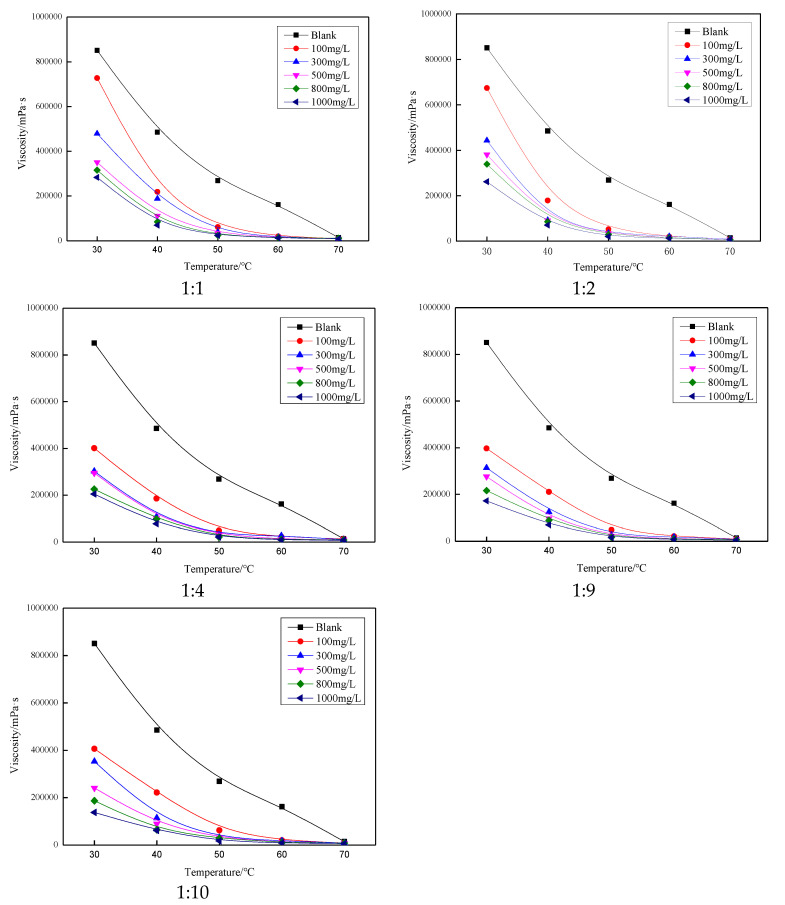
Effect of concentration on viscosity of oil samples at different volume ratios of modified polystyrene to diesel.

Five crude oil flow improvers obtained by dissolving modified polystyrene in diesel oil at different volumes were subjected to the various concentrations to compare the effect of viscosity reduction. From Figure 6, it can be seen that the crude oil flow improvers at different volumes had a significant influence on the viscosity reduction of Henan oil samples. When the temperature is below 50 °C, the viscosity of this oil sample is not only influenced by the viscosity reducer concentration, but also more significantly by the temperature. When the temperature is higher than 50 °C, the crude oil viscosity decreased significantly. Further was observed that the crude oil flow improver had a significantly lower viscosity reduction effect on Henan oil samples [14]. The viscosity reduction effect of all five crude oil flow improvers increased with increasing concentration of flow improver.

### 3.5. Pour Point Depressing on Henan Oil

The effect of crude oil flow improvers was shown in Table 3. As seen in Table 3, the pour point gradually decreases as the crude oil flow improver concentration increases. When the volume ratio of modified polystyrene to diesel reaches 1:4, the pour point of samples gradually decreases with increasing the volume of diesel. In this case, the different between 0–1000 mL/L concentrations of oil-soluble solutions on the pour point represents 8.8 °C.

### 3.6. Wax Crystal Microscope Analysis

**Figure 7:**
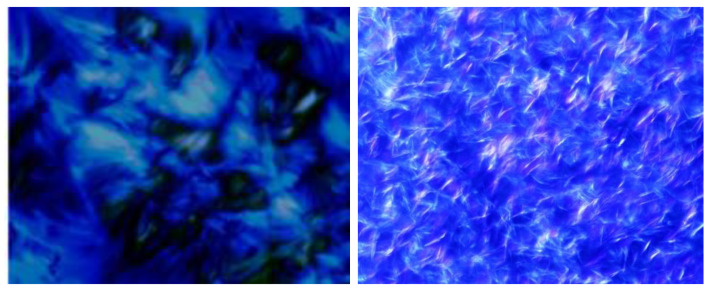
Wax crystals in saturated hydrocarbon without (**left**) and with flow improver (**right**).

The influence of modified polystyrene on the morphology of wax crystal in saturated hydrocarbon of Henan Oilfield sample with 1000 mg/L modified polystyrene was analyzed by polarized light microscope. As can be seen from Figure 7, the wax crystals morphology after the addition of the modified polystyrene are clearly different from the untreated wax crystals. Without the addition of modified polystyrene, the morphology of paraffin crystals is flocculent, and the cross-linking of wax crystals can form a 3D network structure, thus reducing the fluidity of crude oil. The wax crystals formed also encapsulate the liquid oil, thereby reducing the flow of the crude oil [15,16]. The modified polystyrene treatment resulted in a significant reduction and also thinning of the paraffin crystals when compared to the untreated ones, as well as the formation of an irregular network of wax crystals [17,18].

### 3.7. Mechanism

**Figure 8:**
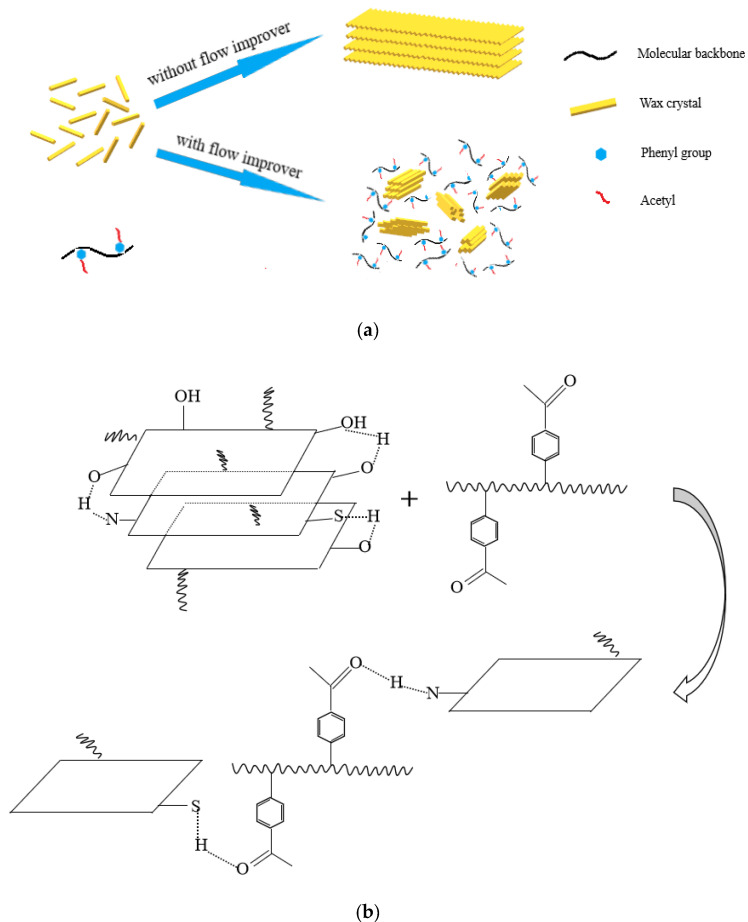
Mechanism of modified polystyrene on the formation of (**a**) the growth mode and (**b**) aggregates formed by overlapping planes wax crystals.

The action mechanism hypothesis of the flow improver is shown in Figure 8. The non-polar groups of the flow improver can interact with the long-chain alkanes, which can be adsorbed on the surface of the wax crystals. Thus, changing the growth mode of the wax crystal and preventing the paraffin from aggregating into larger flaky crystals (as shown in Figure 8a). Furthermore, flow improvers can reduce the viscosity of crude oil by affecting the interaction between colloidal asphaltene molecules. The benzene ring structure contained in the flow improver weakens the supramolecular aggregates formed between gummy asphaltene molecules due to π-π stacking [19,20]. Modified polystyrene contains polar groups that form stronger hydrogen bonds with gum and asphaltene molecules, thus breaking up the aggregates formed by overlapping planes and thus reducing viscosity (as shown in Figure 8b).

## 4. Conclusions

In this paper, waste polystyrene was modified by acetic anhydride to obtain an oil-soluble crude oil flow improver. The reaction product with a molar ratio of styrene to acetic anhydride of 1:1 was dissolved in diesel oil at a volume ratio of 1:10 and the maximum viscosity reduction was achieved at 30 °C with 84.6% viscosity reduction in Henan oil. The addition of crude oil flow improvers also resulted in a significant reduction in the pour point of the oil samples. The morphology analysis of wax crystal shows that the modified polystyrene can co-crystallize with saturated hydrocarbon, which can change the crystallization behavior and orientation of wax and reduce the pour point of the oil sample. Modified polystyrene can interact with gum and asphaltene molecules to change the supramolecular structure in thick oils, thus reducing the viscosity. This article not only proposes the reuse of waste polystyrene plastic, but also provides a solution to the high viscosity of oil samples encountered during crude oil extraction and transportation.

## Figures and Tables

**Table 1:** The basic properties of the crude oil from Henan Oilfield.

Pour Point, °C	Saturated HC, %	Aromatic HC, %	Resin, %	Asphaltene, %
23.5	26.98	28.47	34.12	10.43

**Table 2:** Effect of diesel-pyridine solvent blends on the pour point of oil samples.

**Concentration, mg/L**	0	100	200	300	500	800
**Pour Point,** °C	23.5	22.2	21.8	21.4	21.1	20.7

**Table 3:** Effect of concentration on the pour point of oil samples at the volume ratio of modified polystyrene to diesel.

Concentration (mg/L)	Pour Point				
	1:1	1:2	1:4	1:9	1:10
0	23.5	23.5	23.5	23.5	23.5
100	22.8	21.1	20.6	21.0	21.3
300	21.7	19.0	18.7	20.2	19.0
500	19.6	19.7	17.9	18.6	17.1
800	17.2	17.7	16.2	16.5	15.9
1000	16.3	16.0	14.7	14.9	15.1

## Data Availability

The data presented in this study are available on request from the corresponding authors.
